# An epigenomic roadmap to induced pluripotency reveals DNA methylation as a reprogramming modulator

**DOI:** 10.1038/ncomms6619

**Published:** 2014-12-10

**Authors:** Dong-Sung Lee, Jong-Yeon Shin, Peter D. Tonge, Mira C. Puri, Seungbok Lee, Hansoo Park, Won-Chul Lee, Samer M. I. Hussein, Thomas Bleazard, Ji-Young Yun, Jihye Kim, Mira Li, Nicole Cloonan, David Wood, Jennifer L. Clancy, Rowland Mosbergen, Jae-Hyuk Yi, Kap-Seok Yang, Hyungtae Kim, Hwanseok Rhee, Christine A. Wells, Thomas Preiss, Sean M. Grimmond, Ian M. Rogers, Andras Nagy, Jeong-Sun Seo

**Affiliations:** 1 Genomic Medicine Institute (GMI), Medical Research Center, Seoul National University, Seoul 110-799, Korea; 2 Department of Biomedical Sciences, Seoul National University College of Medicine, Seoul 110-799, Korea; 3 Department of Biochemistry, Seoul National University College of Medicine, Seoul 110-799, Korea; 4 Life Science Institute, Macrogen Inc., Seoul 153-781, Korea; 5 Lunenfeld-Tanenbaum Research Institute, Mount Sinai Hospital, Toronto, Ontario, Canada M5G 1X5; 6 Department of Medical Biophysics, University of Toronto, Toronto, Ontario, Canada M5T 3H7; 7 Faculty of Medical and Human Sciences, University of Manchester, Manchester M13 9PT, UK; 8 Queensland Centre for Medical Genomics, Institute for Molecular Bioscience, The University of Queensland, St Lucia, Queensland 4072, Australia; 9 QIMR Berghofer Medical Research Institute, Genomic Biology Lab, 300 Herston Road, Herston, Queensland 4006, Australia; 10 Genome Biology Department, The John Curtin School of Medical Research, The Australian National University, Canberra, Australian Capital Territory 2601, Australia; 11 Australian Institute for Bioengineering and Nanotechnology, The University of Queensland, Brisbane, Queensland 4072, Australia; 12 Macrogen Bioinformatics Center, Macrogen, Seoul 153-781, Republic of Korea; 13 College of Medical, Veterinary and Life Sciences, University of Glasgow, Glasgow, Scotland G12 8TA, UK; 14 Molecular, Structural & Computational Biology Division, Victor Chang Cardiac Research Institute, Sydney, New South Wales 2010, Australia; 15 Wolfson Wohl Cancer Research Centre, Institute for Cancer Sciences, University of Glasgow, Bearsden, Glasgow Scotland G61 1BD, UK; 16 Department of Physiology, University of Toronto, Toronto, Ontario, Canada M5T 3H7; 17 Department of Obstetrics and Gynaecology, University of Toronto, Toronto, Ontario, Canada M5T3H7; 18 Institute of Medical Science, University of Toronto, Toronto, Ontario, Canada M5T 3H7

## Abstract

Reprogramming of somatic cells to induced pluripotent stem cells involves a dynamic rearrangement of the epigenetic landscape. To characterize this epigenomic roadmap, we have performed MethylC-seq, ChIP-seq (H3K4/K27/K36me3) and RNA-Seq on samples taken at several time points during murine secondary reprogramming as part of Project Grandiose. We find that DNA methylation gain during reprogramming occurs gradually, while loss is achieved only at the ESC-like state. Binding sites of activated factors exhibit focal demethylation during reprogramming, while ESC-like pluripotent cells are distinguished by extension of demethylation to the wider neighbourhood. We observed that genes with CpG-rich promoters demonstrate stable low methylation and strong engagement of histone marks, whereas genes with CpG-poor promoters are safeguarded by methylation. Such DNA methylation-driven control is the key to the regulation of ESC-pluripotency genes, including *Dppa4, Dppa5a* and *Esrrb*. These results reveal the crucial role that DNA methylation plays as an epigenetic switch driving somatic cells to pluripotency.

## Abstract (web summary)

Somatic cell reprogramming can induce distinct pluripotent states. Here the authors perform time-resolved whole-genome bisulfite sequencing during the reprogramming of mouse embryonic fibroblasts and report dynamic global DNA methylation changes.

## Graphical abstract

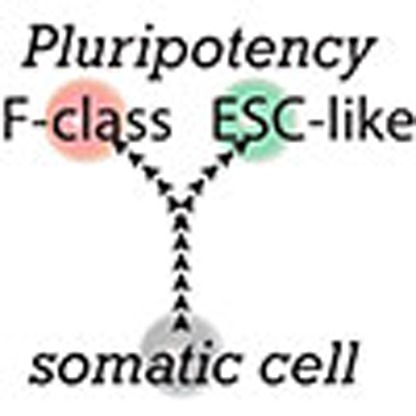

Somatic cells can be reprogrammed into induced pluripotent stem cells (iPSCs) by the expression of defined transcription factors^1^,^2^,^3^,^4^,^5^. During the reprogramming process, the global epigenetic landscape has to be reset to establish the epigenetic marks of the pluripotent state through DNA methylation and chromatin-remodelling processes^2^,^6^,^7^,^8^,^9^. Through the development of a secondary reprogramming system^10^, iPSC generation was initially described as a multistep process characterized by transcriptional, DNA methylation and chromatin changes^11^,^12^,^13^,^14^. Genome-wide analysis of specific chromatin modification dynamics at early stages of reprogramming indicated that this progress might be constrained by repressive epigenetic modifications, such as H3K9me3 and DNA methylation^15^,^16^,^17^,^18^.

More recently, it has been proposed that DNA methylation during iPSC generation functions in the silencing of genes involved in differentiation, while also facilitating chromatin remodelling^18^,^19^,^20^. DNA demethylation appears to play an important role in reactivating pluripotency genes, which are hypermethylated and silenced in somatic cells, particularly in the late stages of the reprogramming process^13^. However, overall understanding of the global dynamics of epigenetic modification at different stages during reprogramming remains poor.

In this work, we have utilized a murine secondary reprogramming system to sample cellular trajectories during reprogramming and performed whole-genome bisulfite sequencing, chromatin immunoprecipitation sequencing (ChIP-seq; H3K4me3, H3K27me3 and H3K36me3), and RNA sequencing (RNA-Seq) to characterize the epigenomic roadmap to pluripotency at base resolution^21^,^22^. Our observations provide a deeper understanding of the reprogramming process and reveal the crucial role that DNA methylation plays in the epigenetic switch that drives somatic cells to pluripotency.

## Results and Discussion

### Dynamic changes in DNA methylation during reprogramming

**Figure 1:**
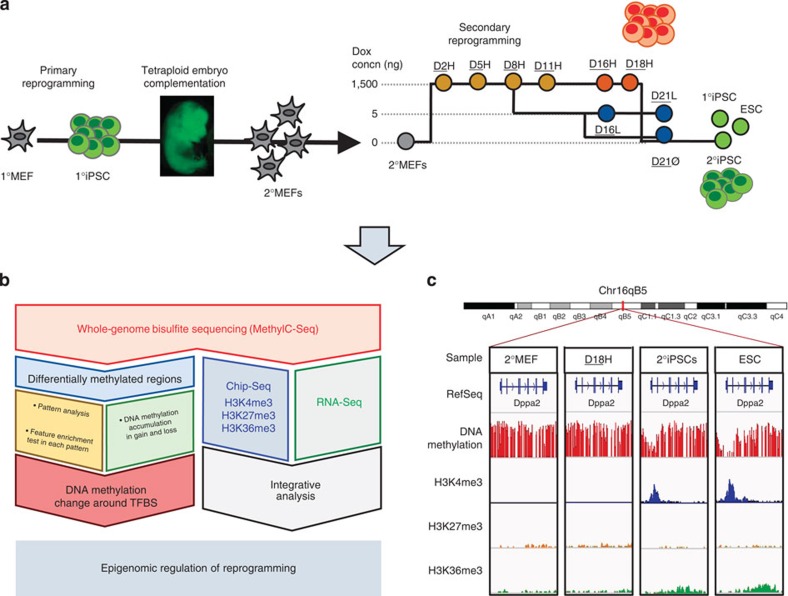
Experimental and computational analysis overview of the study. (**a**) Establishment of secondary system and sample collection. (**b**) MethylC-Seq was performed on samples from secondary system. DMRs were identified. RNA-Seq and ChIP-Seq data were integrated with MethylC-Seq data based on transcripts. (**c**) Base-level visualization of DNA methylation and histone distribution around *Dppa2*.

The Project Grandiose secondary reprogramming samples present a unique opportunity to profile cellular state changes at various time points during reprogramming^10^,^21^,^22^. These consisted of secondary mouse embryonic fibroblasts (2°MEF), six intermediate time points at high doxycycline (dox) concentrations (D2H, D5H, D8H, D11H, D16H and D18H), three alternative intermediate time points collected for samples treated with reduced dox concentrations (D16L, D21L and D21Ø), the secondary iPSCs (2°iPSCs), the primary iPSCs (1°iPSCs) used to generate the chimeric mouse and a mouse Rosa rtTA embryonic stem cell line (ESC) for standard comparison (Fig. 1a–c). As described in ref. 21, these samples showed reprogramming to two distinct pluripotent states: ESC-like cells and the ‘F-class’ consisting of stages D16H and D18H.

**Figure 2:**
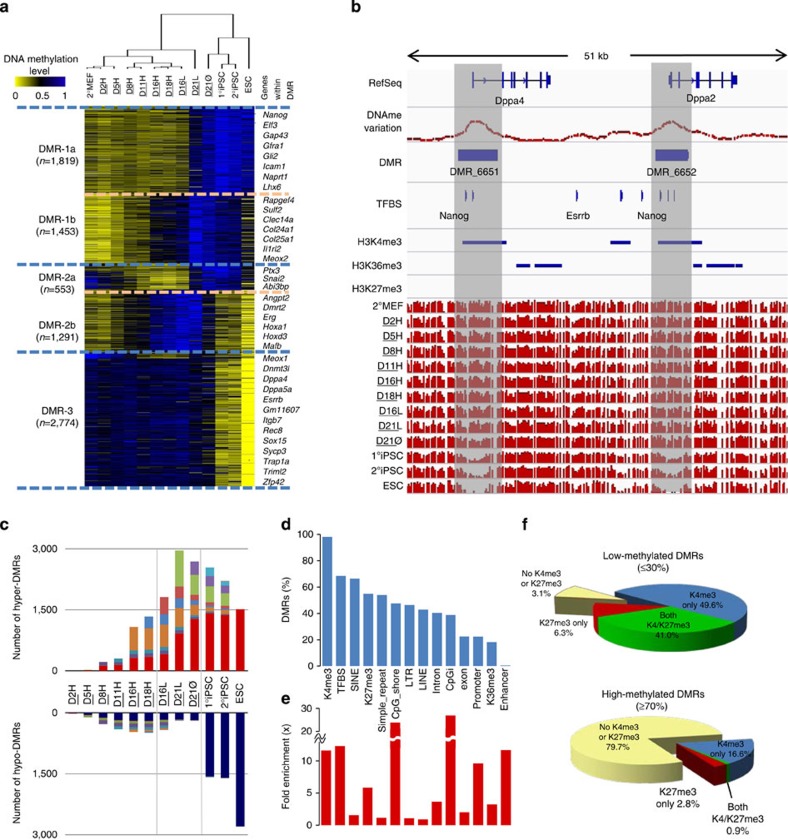
DMRs and features affecting DNA methylation change during reprogramming. (**a**) Hierarchical clustering based on the DNA methylation level of DMRs in each sample. Each DMR was centred with the mean and normalized. DMRs were clustered into six groups based on pairwise correlations. (**b**) Base-level visualization of two DMRs from group DMR-3b in the promoter regions of *Dppa4* and *Dppa2*, known ESC-pluripotency predictor genes. (**c**) DMR accumulation during reprogramming. DMRs were defined as hyper- and hypo-DMRs at each time point. Dark red and dark blue bars represent ESC-specific Hyper- and Hypo-DMRs. Other colours indicate Hyper- and Hypo-DMRs in the order of left to right. (**d**) Proportion of DMRs containing various genomic features. (**e**) Fold enrichment of examined genomic features within DMRs. (**f**) Percentage of DMRs containing H3K4me3 or H3K27me3 based on the methylation level (low-methylated ≤30%, high-methylated ≥70%).

In this manuscript, we describe base-resolution bisulfite sequencing of the 13 Project Grandiose samples and investigation of global DNA methylation changes during reprogramming (Supplementary Data 1). The sample methylomes were scanned using a sliding window of 30 CpGs, identifying 7,890 differentially methylated regions (DMRs) covering 22 Mb, representing 0.81% of the mouse genome (Fig. 2a,b, Supplementary Data 2, Supplementary Figs 1a–c, 6a). Unsupervised hierarchical clustering performed on the DNA methylation state of DMRs (Fig. 2a) distinguished the intermediate states (D2H-D18H and D16L-D21L) from the ESC-like pluripotent states (D21Ø, 1^o^iPSCs, 2^o^iPSCs and ESCs). DMRs were categorized into three groups based on the changing pattern of DNA methylation (Fig. 2a). The DMR-1 group exhibited increased methylation levels after (DMR-1a) or during (DMR-1b) high-level reprogramming factor expression and included genes related to development and cell differentiation, such as the Hox family, *Col25a1* and *Meox2*. The DMR-2 group represented differential methylation changes between two pluripotent states: either gradual demethylation to F-class and methylation in the ESC-like state (DMR-2a) or gradual methylation to F-class and acquired demethylation in the ESC-like state (DMR-2b). A final group (DMR-3) was identified as exhibiting low methylation levels in the ESC-like state (1°iPSCs, 2°iPSCs and ESCs), with stable methylation persisting in the F-class state and intermediate reprogramming samples, which included multiple pluripotency genes such as *Dppa2*, *Dppa4, Dppa5a*, *Esrrb*, *Tcl1* and *Eras* (Fig. 2a, Supplementary Data 2).

We annotated the DMRs in each sample as Hyper- or Hypo-DMRs where they differed from a corresponding 2°MEF baseline by over 20% (Fig. 2c). We observed a widespread gradual increase in methylation to generate Hyper-DMRs during reprogramming, whereas limited demethylation was observed as cells reprogrammed to the F-class state (D16H and D18H). The steady increase in Hyper-DMRs during both high-dox and low-dox reprogramming challenges the notion that most changes in DNA methylation occur at a late stage when cells acquire stable pluripotency^13^. A similar trend was observed for the average methylation level of DMRs, as methylation occurred gradually, while demethylation did not change significantly during transgene expression (Supplementary Fig. 2a,b). Almost all Hypo-DMRs found in iPSCs were also observed in ESCs (98.94%); however, this was not the case for Hyper-DMRs (61.88%), suggesting that demethylation during reprogramming occurred more conservatively.

### TFBSs and histone modification are enriched in the DMRs

To assay the distribution of histone marks, we performed ChIP-Seq for H3K4me3, H3K27me3 and H3K36me3 (see Methods). We determined the distribution and enrichment of these histone marks within DMRs, as well as other genomic features including ESC-TFBSs from published data^23^,^24^,^25^,^26^ (Supplementary Data 3). Notably, we found that 98% of DMRs contained H3K4me3 clusters and 68% contained ESC-TFBSs (Fig. 2d). When we assessed enrichment of each feature relative to the whole genome, H3K4me3 marks, ESC-TFBSs, CpG islands, CpG shores and enhancers showed more than 10-fold enrichment, followed by promoters and H3K27me3 clusters (Fig. 2e).

Our finding that histone marks were highly enriched within DMRs led us to explore the relationship between DNA methylation levels and H3K4me3/H3K27me3 marks within DMRs (Fig. 2f, Supplementary Fig. 2c, Supplementary Table 1). DMRs exhibiting low-level methylation (less than 30%) were frequently associated (96.9%) with H3K4me3 and H3K27me3. In contrast, the absence of both histone marks was most frequently associated (79.7%) with DMRs with high levels of methylation (≥70%), supporting the inverse relationship between DNA methylation and these two histone modifications. Furthermore, CpGs inside H3K4me3 and H3K27me3 marks exhibit more methylation change, in comparison with CpGs inside H3K36me3 mark (Supplementary Fig. 2d).

To investigate the involvement of ESC-TFBSs in reprogramming, we performed separate enrichment analysis for each DMR group (Table 1). Polycomb-repressive complex (PRC)-binding sites, including SUZ12, EZH2 and RING1B, were enriched in DMR-1 and DMR-2b. On the other hand, sequence-specific pluripotency-associated ESC-TFBSs such as Nanog, Oct4 and Klf4 (but not CTCF and TET1)-binding sites were enriched in DMR-3, the group of DMRs that are demethylated only in the ESC-like state. These results demonstrate the dynamic changes in DNA methylation at TFBSs, and the connection between the pattern of changes and TFBS enrichment.

### Dynamic changes of TFBS methylation during reprogramming

**Figure 3:**
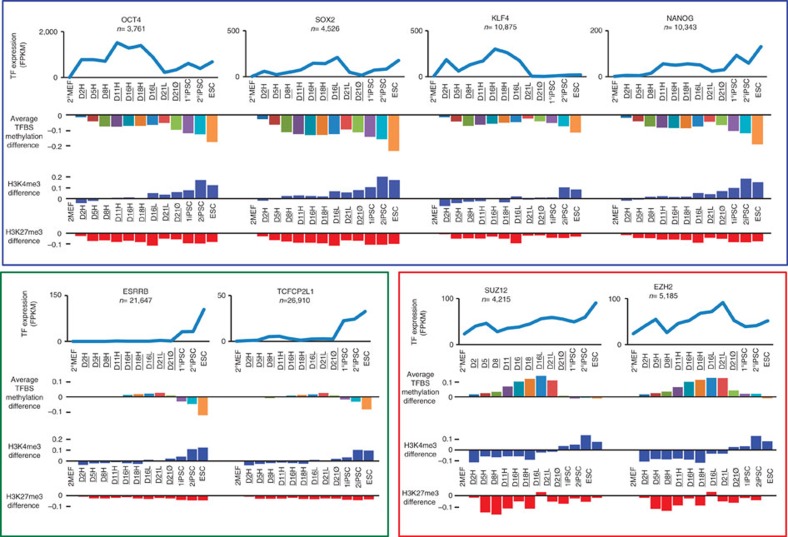
Histone modification and DNA methylation change at transcription factor-binding sites. RNA expression level (FPKM) of transcription factors (line plots), average DNA methylation change (upper bar plots), average H3K4me3 change (blue bar plots) and average H3K27me3 change (red bar plots) at binding sites of each transcription factor. Selected transcriptionally active genes during high-dox treatment (blue box), transcriptionally silent genes during high-dox treatment (green box) and polycomb repressive complexes (red box) are shown.

Interrogating methylation changes at ESC-TFBSs resulted in the detection of methylation depletion during high-dox treatment, which was not apparent by examining DMRs (Fig. 3, Supplementary Fig. 3; Methods). This was most obvious at the binding sites for activated or overexpressed transcription factors during early time points, such as OCT4, SOX2, KLF4 and NANOG. These TFBSs also accumulated H3K4me3 modifications that proceed after the methylation depletion. H3K27me3 marks diminished at binding sites of expressed transcription factors early in reprogramming. In contrast, ESC-TFBSs for genes that were not activated during high-dox reprogramming but are known to play critical roles in ESC-like pluripotent state, such as ESRRB and TCFCP2L1 (refs 14, 27, 28), showed no change in DNA methylation and were demethylated only in the ESC-like state. The PRC (SUZ12 and EZH2)-binding sites underwent a gain of DNA methylation during reprogramming but showed baseline levels of methylation in ESC.

**Figure 4:**
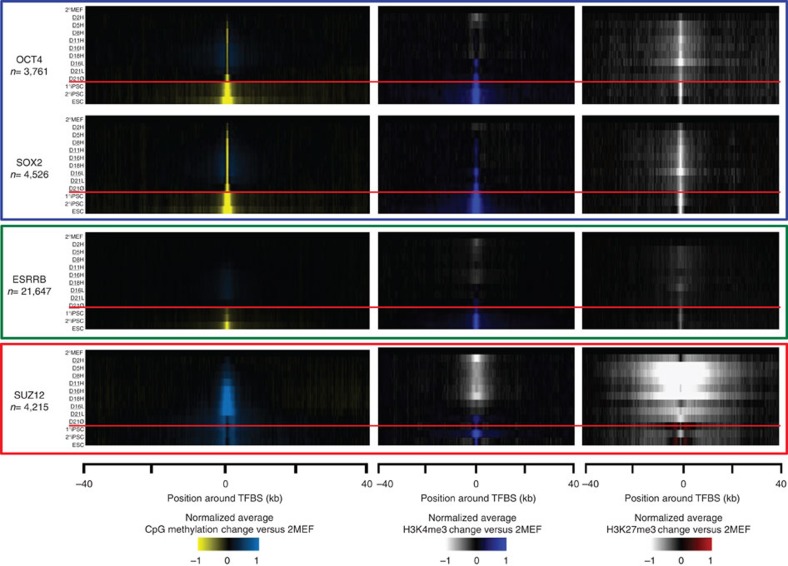
Histone modification and DNA methylation change around transcription factor-binding sites. Average DNA methylation change (left), average H3K4me3 change (middle) and average H3K27me3 change (right) in the 80-kb neighbourhood of transcription factor-binding sites.

We assessed DNA methylation changes occurring within ±40 kb of ESC-TFBSs (Fig. 4, Supplementary Fig. 4). At the binding sites of core ESC-pluripotency transcription factors (OCT4, SOX2, KLF4 and NANOG), we observed rapid focal demethylation during high-dox treatment (D2H-D18H) if the factors were expressed. On the other hand, ESC-like cells (1°iPSC, 2°iPSC and ESC) exhibited extensive demethylation, up to 20 kb distal from the binding sites. A similar but more delayed process was also observed for H3K4me3 modifications. The broad neighbourhoods around PRC-binding sites were hypermethylated in all samples examined. Interestingly, although methylation accumulated broadly around PRC (SUZ12, EZH2 and RING1B)-binding sites (Fig. 4, Supplementary Fig. 4), these underwent focal renormalization at the ESC-like pluripotent state. These sites also demonstrate bivalent marks of H3K4me3 and H3K27me3 in ESC-like state^24^. The patterns of change to DNA methylation and histone marks were distinct for the three types of transcription factor shown (Figs 3 and 4). Our results show an interesting contrast between the focal demethylation induced early in reprogramming and broader demethylated regions at ESC-like pluripotent state, perhaps representing a key distinguishing feature of the pluripotent state where broader demethylation is required for completion of the reprogramming to ESC-like state.

We attempted to show that the dynamics of methylation change at transcription factor-binding sites (TFBSs) could act as a predictor of importance to the reprogramming process. We proposed criteria for DNA-binding transcription factors of >1.2 × enrichment and >10% overlap in DMR-3, implying over-representation in DMRs that underwent demethylation at transition to the ESC-like state, but little change early in reprogramming. We tested a set of 118 transcription factors with computationally predicted binding sites against these criteria^29^,^30^. We found only three transcription factors (SOX2, MYC and OCT4) that fulfilled our criteria, all of which are known to be important in reprogramming to iPSCs (Supplementary Data 4). This suggests a high specificity for the prediction criteria, although sensitivity is low as other factors known to be involved in reprogramming were not identified. Transcription factors whose binding sites show significant change in methylation late in a transition can be called important to that transition with high confidence. We believe that methylome-based tests of this nature could have useful application in prediction of transcription factors involved in other cellular transitions.

### Demethylation leads to precise control of gene expression

We integrated corresponding RNA expression data^22^ with our DNA methylation and histone modification data sets (Supplementary Tables 2–4, Supplementary Data 5; Methods). Activation of genes was associated with H3K4me3 occupancy in promoter regions and repression was associated with either H3K27me3 occupancy or no histone mark (Supplementary Fig. 5a). Moreover, as we observed in DMRs, engagement of both H3K4me3 and H3K27me3 marks in promoters was dependent on DNA methylation levels with a strong inverse relationship (Supplementary Fig. 5b).

**Figure 5:**
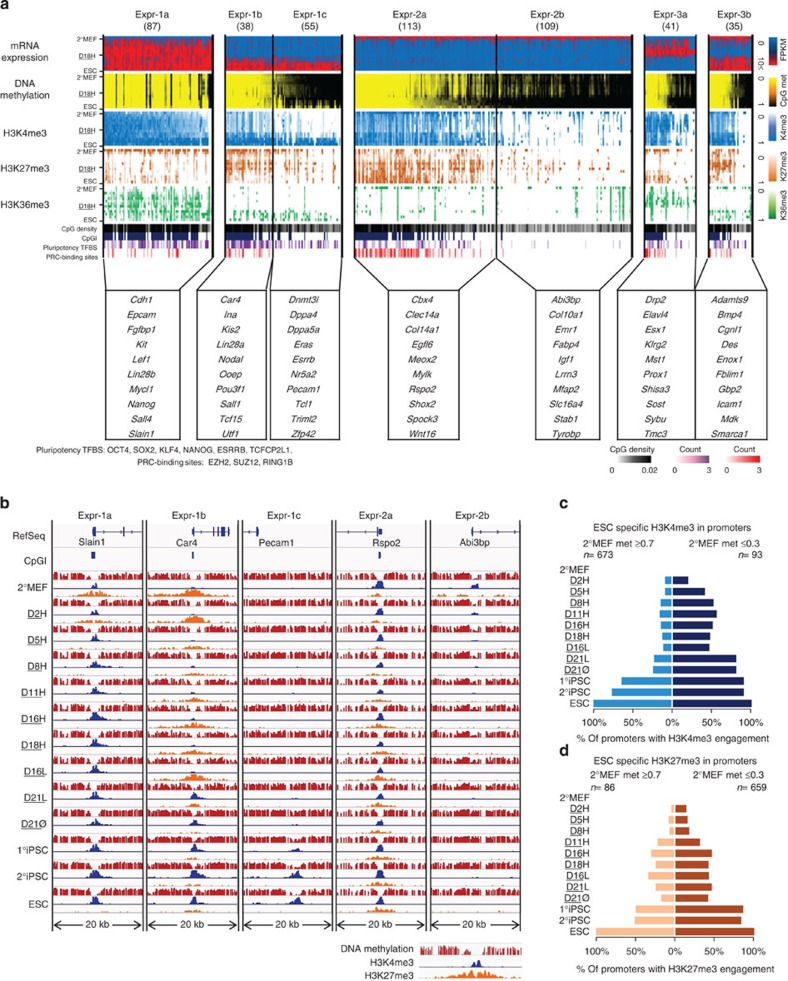
Epigenetic features of gene classes and model of gene expression control. (**a**) Genes were separated into clusters based on gene expression patterns and DNA methylation. The heatmap presents mRNA expression, DNA methylation level of promoter regions, normalized H3K4me3 level, normalized H3K27me3 level, CpG densities, pluripotency transcription factor-binding sites and binding sites of PRCs. (**b**) Base-level visualization of DNA methylation and histone modifications in the promoter regions of representative genes for each class across all samples. (**c**) Percentage of ESC-specific H3K4me3 mark for promoters with high and low initial methylation. (**d**) Percentage of ESC-specific H3K27me3 mark for promoters with high and low initial methylation.

We selected 477 genes segregating into seven clusters on the basis of expression and epigenetic change over the course of reprogramming (Fig. 5a,b, Supplementary Table 5; Methods). These groups represent: activated early in reprogramming (Expr-1a), activated late in reprogramming with either low- (Expr-1b) or full- (Expr-1c) DNA methylation in 2°MEF and repressed during reprogramming with either low- (Expr-2a) or full- (Expr-2b) DNA methylation in ESC. Genes in Expr-3a were turned on, while those in Expr-3b were turned off in high-dox; therefore, they were differentially expressed between D16H/D18H (F-class cells) and ESC-like cells. Expression changes of genes in Expr-1a and Expr-2a/b are likely responsible for pluripotency, as they were differentially expressed between 2°MEF and pluripotent cells^21^. Finally, the presence of genes in Expr-1b/c explains why F-class cells are distinct from ESC-like state cells.

The expression dynamics through reprogramming of these genes was clear upon visualization of the categories and representative genes from each class (Fig. 5a,b, Supplementary Fig. 5a–d). Genes repressed by H3K27me3 with low-methylated promoters in 2°MEF tended to be activated early in reprogramming and had CpG-rich promoters (Expr-1a/b). These loci were enriched in genes involved in cell adhesion, such as *Epcam* and *Cdh1* (Fig. 5a (Expr-1a)). In contrast, quiescence of Expr-1c genes was initially safeguarded by DNA methylation of CpG-poor promoters, and H3K4me3 was only acquired after late demethylation. The same two modes of control were observed for the genes repressed by reprogramming. However, as in the analysis of DMRs, DNA methylation in promoter regions happened early in reprogramming (Expr-2b), whereas demethylation was detected exclusively in the ESC-like state, revealing that a gain of methylation is kinetically favoured over demethylation. This is also true for histone marks in relation to changes in gene expression, where histone modifications, specifically the modulation of H3K27me3, occurred early during reprogramming (Expr-2a) within low-methylated promoters. Interestingly, the dynamic process of histone modification alterations during reprogramming was strongly influenced by the starting methylation state of gene promoters (Fig. 5c,d). Genes with low-methylated promoters at 2°MEF showed a significantly higher rate of transition to the ESC-like state for both ESC-specific histone marks compared with those with fully methylated promoters. This suggests that DNA methylation presents a major barrier during somatic cell reprogramming to ESC-like cells and that the methylation status of a given region determines its control by histone modifications.

**Figure 6:**
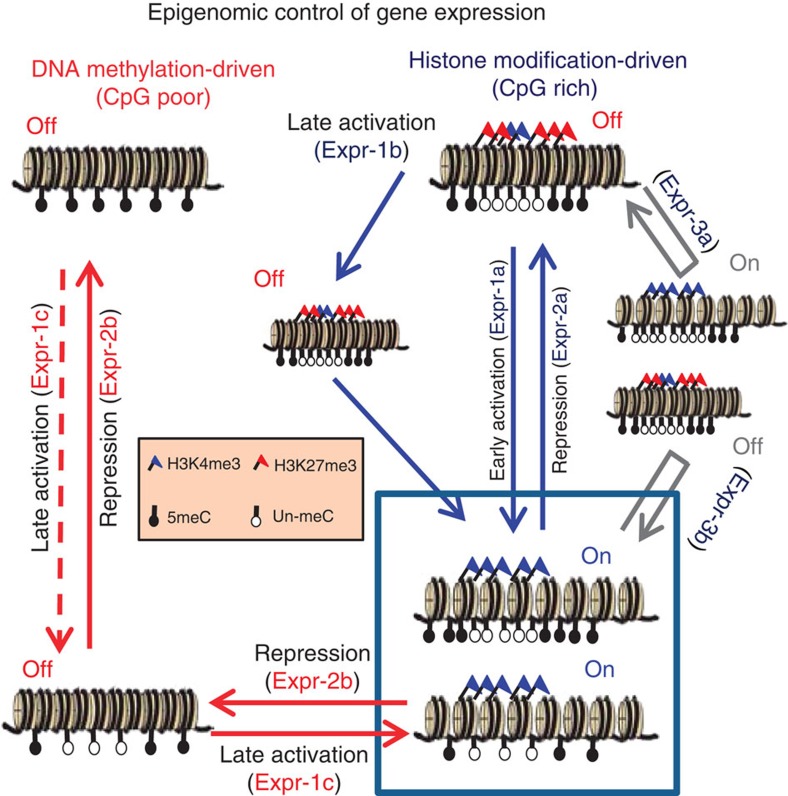
A model summarizing DNA methylation and histone modification-driven control of gene expression. Dashed arrow represents the strict control of demethylation. Gene classes affected by changes are shown in brackets accompanying arrows.

We propose a model that describes the key mechanism of epigenetic control of gene expression during reprogramming (Fig. 6). In genes with CpG-poor promoters, control is driven by DNA methylation. Such genes may be activated by demethylation followed by H3K4me3 engagement, producing expression profiles characteristic of class Expr-1c/2b. In genes with CpG-rich promoters, low methylation levels allow histone modification-driven control. This model is supported by data showing the role of initial methylation status as a modulator of the dynamic changes to histone modification, and the sequential modification of DNA methylation followed by histone marks in TFBSs. The model also accounts for characteristic gene expression classes (detailed in Figs 5 and 6). We predict that this mechanism may not only apply to iPSC reprogramming but also to lineage specification of cells. Therefore, our insights into how DNA methylation controls the epigenetic landscape in reprogramming to pluripotency could be crucial to a better understanding of the mechanisms underlying general cell fate change, and could have ramifications for stem cell-based therapies.

## Methods

### Cell culture and secondary reprogramming

ROSA26-rtTA-IRES-GFP mouse ESC, iPSCs and mouse embryonic fibroblasts were cultured as previously described^31^. ESCs and iPSCs were cultured in 5% CO_2_ at 37 °C on irradiated MEFs in DMEM containing 15% FCS, leukaemia-inhibiting factor, penicillin/streptomycin, L-glutamine, nonessential amino acids, sodium pyruvate and 2-mercaptoethanol. 1B 1° iPS cells were aggregated with tetraploid host embryos as described^10^ and MEFs established from E13.5 embryos. High-dox cell samples were collected on days 0, 2, 5, 8, 11, 16 and 18 (D2H, D5H, D8H, D11H, D16H and D18H). A subculture of the reprogramming cells was established from day 19 and cultured in the absence of dox, to develop a factor-independent 2° iPS cell line by day 30 (2°iPSC). Low-dox samples were maintained from day 8 to day 14 cells in 5 ng dox. On day 14 the culture was diverged into two, with some of the cells being cultured until day 21 in the absence of dox (D21Ø) and the remainder being cultured in 5 ng ml^−1^ of dox and collected on day 16 (D16L) and (D21L). Rosa26rtTA ESCs and 1B 1o iPSCs were collected as controls.

### MethylC-Seq library generation

For all 13 samples (2°MEF, D2H, D5H, D8H, D11H, D16H, D18H, D16L, D21L, D21Ø, 1°iPSC, 2°iPSC and rtTA ESC), 5 mg of genomic DNA was mixed with 25 ng unmethylated cl857 Sam7 Lambda DNA (Promega, Madison, WI, USA). The DNA was fragmented by sonication to 300–500 bp with a Covaris S2 system (Covaris) followed by end repair with the End-It DNA End-Repair Kit (Epicenter). Paired-end universal library adaptors provided by Illumina were ligated to the sonicated DNA as per the manufacturer’s instructions for genomic DNA library construction. Ligated products were purified with AMPure XP beads (Beckman, Brea, CA, USA). Adaptor-ligated DNA was bisulfite-treated using the EpiTect Bisulfite Kit (QIAGEN) following the manufacturer’s instructions and then PCR-amplified using PfuTurboCx Hotstart DNA polymerase (Agilent, Santa Clara, CA, USA) with the following PCR conditions (2 min at 95 °C, 4 cycles of 15 s at 98 °C, 30 s at 60 °C, 4 min at 72 °C and then 10 min at 72 °C). The reaction products were purified using the MinElute gel purification kit (QIAGEN). The sodium bisulfite non-conversion rate was calculated as the percentage of cytosines sequenced at cytosine reference positions in the lambda genome.

### ChIP library generation

ChIP was carried out as described in ref. 32. In all, 40–150 million cells were fixed with 1% formaldehyde for 10 min at room temperature, and scraped and stored as pellets (−80 °C). Samples were lysed at 20 million cells per ml Farnham lysis buffer for 10 min and subsequently at 10 million cells per ml nuclear lysis buffer. The released chromatin was sheared to 100–500 bp (250 bp average) on ice using a SonicsVibraCell Sonicator equipped with a 3-mm probe. For each sample, 50 μl of solubilized chromatin was used as input DNA to normalize sequencing results and the remaining chromatin was immunoprecipitated with 10 μg of H3K4me3 (ab8580)^33^, 10 μg H3K27me3 (Millipore 07-449)^16^ or 10 μg H3K36me3 (ab9050)^16^ antibodies, separately. Antibody–chromatin complexes were pulled down with 100 μl magnetic Protein G Dynal beads (Invitrogen) and washed six times. The chromatin was then eluted, reverse crosslinked at 65 °C overnight and subjected to RNaseA/proteinase K treatment. ChIP and input DNA were purified using a Qiagen Purification Column and quantified using a Quant-it dsDNA High Sensitivity Assay (Invitrogen). For ChIP sequencing, ChIP-seq libraries were prepared according to the protocols described in the Illumina ChIP-seq library preparation kit. Briefly, 50 ng of immunopurified DNA or 100 ng of genomic DNA from an input sample was end-repaired, followed by the 3′ addition of a single adenosine nucleotide and ligation to universal library adapters. Ligated material was separated on a 2.0% agarose gel, followed by the excision of a 250- to 350-bp fragment and column purification (QIAGEN). DNA libraries were prepared by PCR amplification (18 cycles).

### High-throughput sequencing

MethylC-Seq DNA and ChIP DNA libraries were sequenced using the Illumina HiSeq 2000 as per the manufacturer’s instructions. Sequencing of libraries was performed up to 2 × 101 cycles. Image analysis and base calling were performed with the standard Illumina pipeline version RTA 2.8.0.

### Processing and alignment of MethylC-Seq data

MethylC-Seq sequencing data were processed using the Illumina analysis pipeline, and FastQ format reads were aligned to the NCBI37/mm9 mouse reference using the Bismark/Bowtie alignment algorithm^18^,^34^,^35^. Paired-read MethylC-Seq sequences produced by the Illumina pipeline in FastQ format were trimmed with trim threshold 1,500; we removed the last two bases from sequences that were not trimmed and removed three bases from sequences that were trimmed. The Bismark package version 0.7.7 was used as the aligner using the following parameters: -e 90 -n 2 -l 32 -X 550. As up to six independent libraries from each biological replicate were sequenced, we first removed duplicate reads. Subsequently, the reads from all libraries of a particular sample were combined. Unique read alignments were then subjected to post-processing. The number of calls for each base at every reference sequence position and on each strand was calculated. All results of aligning a read to both the Watson and Crick converted genome sequences were combined. The CpG methylation levels were calculated using bisulfite conversion rates by (Number of not converted Cs per read depth) for each position (Supplementary Data 1).

### RNA-Seq library generation and sequencing

Total RNA was subjected to two rounds of on column DNAseI treatment to remove contaminating DNA using the RNase-Free DNase set (Qiagen PN 79254) as per the manufacturer’s protocol. The total RNA was then analysed using the Agilent RNA 6000 Nano Kit (PN 5067-1511) on the Agilent Bioanalyzer 2100 (PN G2939AA) to quantify yield, qualify integrity and confirm removal of DNA contamination.

Following DNAseI treatment, 5 μg total RNA from each sample was depleted of ribosomal RNA using the Ribo-ZerorRNA Removal Kit (Epicenter PN RZH110424) as per the manufacturer’s instructions. The ribosomal-depleted RNAs were then run on an Agilent RNA 6000 Pico Kit (PN 5067-1513) on the Agilent Bioanalyzer 2100 to confirm ribosomal RNA depletion. Sequencing libraries where generated from the ribosomal-depleted RNA using the SOLiD Transcriptome Multiplexing Kit (PN 4427046) from Applied Biosystems following the manufacturer’s publication. Final libraries were quantified and qualified using the Agilent High Sensitivity DNA Kit (PN 5067-4626) on the Agilent Bioanalyzer 2100.

Sequencing libraries were subsequently pooled in equimolar ratios (four libraries per pool) and clonally amplified on SOLiD nanobeads. Clonal amplification was completed via emulsion PCR using the SOLiD EZ Bead System (PN 4448419, 4448418 and 4448420) coupled with SOLiD EZ Bead N200 amplification reagents (PN 4467267, 4457185, 4467281, 4467283 and 4467282). Following emulsion PCR, clonally amplified nanobeads were enriched using the SOLiD EZ Bead Enricher Kits (PN 4467276, 4444140 and 4453073) before being deposited into SOLiD 6-Lane FlowChip (PN 4461826) using the SOLiD Flowchip Deposition Kit v2 (PN 4468081) as per the manufacturer’s recommendations.

In total, two flowchips were sequenced yielding a total of eight lanes of data, with sequencing reads generated using the SOLiD 5500xl platform generating paired 75 bp forward and 35 bp reverse reads. To allow de-convolution of the pooled libraries, a single 5-bp index read was generated. A total of 1,204,676,394 fragments (2,409,352,788 reads) were generated post deconvolution, ranging from 35,714,748 to 147,282,580 fragments per library.

### Processing and alignment of RNA-Seq data

Sequence mapping was performed using Applied Biosystems LifeScope v2.5 whole transcriptome (paired-end) analysis pipeline against the NCBIM37 (mm9) genome and exon-junction libraries constructed from the Ensembl v64 gene model. Briefly, this pipeline first removes potential contaminant reads by aligning to a filter set containing rRNA, tRNA, adaptor sequences and retrotransposon sequences. Following filtering, LifeScope then aligns all reads to the genome and F3 reads to the junction library. F5 reads are additionally aligned at a higher sensitivity to exonic sequences within insert size distance from the paired (F3) read alignment. Read alignments are merged and disambiguated, and a single BAM (binary alignment/mapped) file output per library.

BAM files were then additionally filtered to remove reads with a mapping quality (MAPQ)<9 and all mitochondrial reads. Alignments were then assembled using Cufflinks (v2.0.2) using the –G parameter to quantify gene and isoform FPKM expression values against the reference gene model (Ensembl v67).

### Identification of methylated cytosines

At each reference cytosine, the binomial distribution was used to identify whether at least a subset of the genomes within the sample were methylated, using a 0.01 FDR-corrected *P* value. We identified methyl cytosines while keeping the number of false-positive methylcytosine calls below 1% of the total number of methyl cytosines we identified. The probability *P* in the binomial distribution *B*(*n*, P) was estimated from the number of cytosine bases sequenced in reference cytosine positions in the unmethylated Lambda genome (referred to as the error rate: nonconversion plus sequencing error frequency). We interrogated the sequenced bases at each reference cytosine position one at a time, where read depth refers to the number of reads covering that position. For each position, the number of trials (*n*) in the binomial distribution was the read depth. For each possible value of *n* we calculated the number of cytosines sequenced (*k*) at which the probability of sequencing *k* cytosines out of *n* trials with an error rate of *p* was less than the value *M*, where M* (number of unmethylated cytosines) <0.01* (number of methylated cytosines) and if the error rate of *p* was over 0.01, we assumed that the cytosine was not methylated. In this way, we established the minimum threshold number of cytosines sequenced at each reference cytosine position at which the position could be called as methylated, so that out of all methyl cytosines identified no more than 1% would be because of the error rate.

### Calculation of DNA methylation level

If the error rate is less than 0.01 we calculated adjusted DNA methylation level for cytosine as follow:


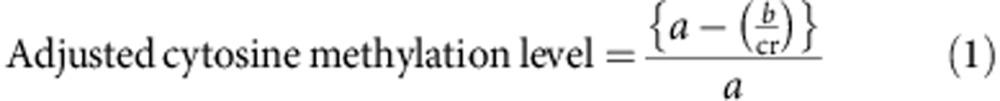


(*a*=total Cs, *b*=number of converted Cs, cr=bisulfite conversion rate).

### Identification of DMRs

DMRs (Fig. 2) were identified using a sliding window approach (Supplementary Fig. 6a, Fig. 2b). A window size of 30 CpGs less than 6 kb with coverage more than 5 × in 15 CpGs per window in all samples were considered, progressing one CpG per iteration. Total of 20,214,978 windows were assessed. Windows showing maximum difference and fold enrichment of 30% and fourfold with Benjamini–Hochberg-corrected FDR from analysis of variance (ANOVA) test *P* values of less than 1% were identified as differentially methylated windows. In all, 188,529 differentially methylated windows were then joined if regions were overlapped or progressing region and the succeeding regions were covering more than 60% of the region. This set of 7,890 DMRs covering 21,618,964 bp of the whole genome are reported in Fig. 2 and Supplementary Data 2.

DMRs were then defined as Hyper-DMRs and Hypo-DMRs if the average methylation level difference of each DMR in each sample was higher or lower by more than 20% relative to 2°MEF.

### Mapping and enrichment analysis of ChIP-Seq reads

Paired-end ChIP-Seq data were processed using the Illumina analysis pipeline, and mapping was conducted using Bowtie version 0.12.8 with the following parameters: --pairtries 100 -y -k 1 -n 3 -l 50 -I 0 -X 1000. Enrichment analysis was conducted using MACS^36^ with parameters of --nomodel -S -w –n –space 30.

### ChIP-Seq data analysis

Enriched peaks from ChIP-Seq data were joined into clusters where at least one sample has a peak for each modification (H3K4me3, H3K27me3 and H3K36me3; Supplementary Fig. 6b). The total peak width of each sample within the cluster was calculated as histone mark score within clusters.

### TFBS epigenomic change analysis

ESC-TFBSs of mouse ESCs were obtained from different studies^23^,^24^,^25^. CpG methylation level of each TFBS in each sample was calculated. The average CpG methylation change of each TFBS was than calculated in each sample relative to 2°MEF (Fig. 3). For calculating CpG methylation change around ESC-TFBSs, the same procedure was applied for 200 bp 400 bins around each ESC-TFBS. The same procedure using enrichment score for 30-bp window was applied for calculating average histone modification change (Fig. 4).

### Genome annotation

Genomic regions and CpG islands were defined based on NCBI37/mm9 coordinates downloaded from the UCSC website (http://genome.ucsc.edu/). Promoters were arbitrarily defined as 5 kb upstream and 1 kb downstream of transcriptional start site for each Ensembl release-67 transcript. Gene bodies are defined as from transcription start to end sites for each transcript. Histone modification clusters and DMRs were annotated if they overlap with their promoters.

### Fold-enrichment test

Fold enrichment was calculated as follows: (Observed number of *X* in examining region/total length of examining region (bp))/(total number of *X* in reference region/reference region length (bp)), *X*=genomic feature)).

### Gene expression pattern separation

We selected genes of expression patterns as described in Supplementary Table 5.

### Data integration and normalization

DNA methylation levels of promoters were calculated from 5 kb upstream and 1 kb downstream of the transcription start site. H3K4me3 and H3K27me3 marks were considered if their cluster of peaks were overlapped with promoters. Overlapped H3K36me3 peaks were calculated for whole gene. In Fig. 5, for calculating normalized histone modification scores, maximum peak width was considered as 1 and relative widths were calculated for each sample in each gene.

### Accession codes

Methylome sequencing data are available under the European Nucleotide Archive accessions no. ERP004116 (http://www.ebi.ac.uk/ena/data/view/PRJEB4795). Long RNA-seq and Chip-seq sequencing data are available under the NCBI Sequence Read Archive (SRA) accessions no. SRP046744 (http://www.ncbi.nlm.nih.gov/sra). Analysed data sets can be obtained from Stemformatics (www.stemformatics.org)^37^.

## Author contributions

J.-S.S. and A.N. conceived and designed the experiments. J.-Y.S., J.-Y.Y., J.K., K.-S.Y. and H.K. performed MethylC-Seq and ChIP-Seq experiments. P.D.T derived iPSC lines. M.C.P., M.L., S.M.I.H. and I.M.R. performed pull downs for ChIP-Seq. N.C. and S.M.G. performed RNA-Seq. D.-S.L. performed sequencing data processing. D.-S.L., S.L., W.-C.L. and H.R. conducted bioinformatic and statistical analyses. J.-S.S., D.-S.L., J.-Y.S., H.P., T.B. and J.-H.Y. wrote the manuscript.

## Additional information

**How to cite this article:** Lee, D.-S. *et al*. An epigenomic roadmap to induced pluripotency reveals DNA methylation as a reprogramming modulator. *Nat. Commun.* 5:5619 doi: 10.1038/ncomms6619 (2014).

## Supplementary Material

Supplementary Information Supplementary Figures 1-6 and Supplementary Tables 1-5

Supplementary Data 1 MethylC-Seq data set details and alignment summary.

Supplementary Data 2 Number of Transcription factor binding sites

Supplementary Data 3 Counts and enrichment over whole genome and total DMRs of features overlap with each DMR pattern

Supplementary Data 4 Percentages of regions containing predicted binding sites in each DMR group and enrichment over total DMRs.

Supplementary Data 5 Total gene expression, epigenomic status, and number of transcription factor binding sites

## Figures and Tables

**Table 1:** Enrichment of TFBSs in each DMR group.




## References

[b1] Takahashi K. & Yamanaka S. Induction of pluripotent stem cells from mouse embryonic and adult fibroblast cultures by defined factors. Cell 126, 663–676 (2006). 16904174 10.1016/j.cell.2006.07.024

[b2] Maherali N. . Directly reprogrammed fibroblasts show global epigenetic remodeling and widespread tissue contribution. Cell Stem Cell 1, 55–70 (2007). 18371336 10.1016/j.stem.2007.05.014

[b3] Takahashi K. . Induction of pluripotent stem cells from adult human fibroblasts by defined factors. Cell 131, 861–872 (2007). 18035408 10.1016/j.cell.2007.11.019

[b4] Yu J. . Induced pluripotent stem cell lines derived from human somatic cells. Science 318, 1917–1920 (2007). 18029452 10.1126/science.1151526

[b5] Park I. H. . Reprogramming of human somatic cells to pluripotency with defined factors. Nature 451, 141–146 (2008). 18157115 10.1038/nature06534

[b6] Kang L., Wang J., Zhang Y., Kou Z. & Gao S. iPS cells can support full-term development of tetraploid blastocyst-complemented embryos. Cell Stem Cell 5, 135–138 (2009). 19631602 10.1016/j.stem.2009.07.001

[b7] Zhao X. Y. . iPS cells produce viable mice through tetraploid complementation. Nature 461, 86–90 (2009). 19672241 10.1038/nature08267

[b8] Onder T. T. . Chromatin-modifying enzymes as modulators of reprogramming. Nature 483, 598–602 (2012). 22388813 10.1038/nature10953 PMC3501145

[b9] Singhal N. . Chromatin-remodeling components of the BAF complex facilitate reprogramming. Cell 141, 943–955 (2010). 20550931 10.1016/j.cell.2010.04.037

[b10] Woltjen K. . piggyBac transposition reprograms fibroblasts to induced pluripotent stem cells. Nature 458, 766–770 (2009). 19252478 10.1038/nature07863 PMC3758996

[b11] Samavarchi-Tehrani P. . Functional genomics reveals a BMP-driven mesenchymal-to-epithelial transition in the initiation of somatic cell reprogramming. Cell Stem Cell 7, 64–77 (2010). 20621051 10.1016/j.stem.2010.04.015

[b12] Mikkelsen T. S. . Dissecting direct reprogramming through integrative genomic analysis. Nature 454, 49–55 (2008). 18509334 10.1038/nature07056 PMC2754827

[b13] Polo J. M. . A molecular roadmap of reprogramming somatic cells into iPS cells. Cell 151, 1617–1632 (2012). 23260147 10.1016/j.cell.2012.11.039 PMC3608203

[b14] Buganim Y. . Single-cell expression analyses during cellular reprogramming reveal an early stochastic and a late hierarchic phase. Cell 150, 1209–1222 (2012). 22980981 10.1016/j.cell.2012.08.023 PMC3457656

[b15] Chen J. . Vitamin C modulates TET1 function during somatic cell reprogramming. Nat. Genet. 45, 1504–1509 (2013). 24162740 10.1038/ng.2807

[b16] Wang T. . The histone demethylases Jhdm1a/1b enhance somatic cell reprogramming in a vitamin-C-dependent manner. Cell Stem Cell 9, 575–587 (2011). 22100412 10.1016/j.stem.2011.10.005

[b17] Plath K. & Lowry W. E. Progress in understanding reprogramming to the induced pluripotent state. Nat. Rev. Genet. 12, 253–265 (2011). 21415849 10.1038/nrg2955 PMC3273493

[b18] Lister R. . Hotspots of aberrant epigenomic reprogramming in human induced pluripotent stem cells. Nature 471, 68–73 (2011). 21289626 10.1038/nature09798 PMC3100360

[b19] Papp B. & Plath K. Epigenetics of reprogramming to induced pluripotency. Cell 152, 1324–1343 (2013). 23498940 10.1016/j.cell.2013.02.043 PMC3602907

[b20] Surani M. A., Hayashi K. & Hajkova P. Genetic and epigenetic regulators of pluripotency. Cell 128, 747–762 (2007). 17320511 10.1016/j.cell.2007.02.010

[b21] Tonge P. D. . Divergent reprogramming routes lead to alternative stem cell states. *Nature* doi: 10.1038/nature14047 (2014). 10.1038/nature14047 25503232

[b22] Hussein S. M. I. . Genome-wide characterization of the routes to pluripotency. *Nature* doi: 10.1038/nature14046 (2014). 10.1038/nature14046 25503233

[b23] Chen X. . Integration of external signaling pathways with the core transcriptional network in embryonic stem cells. Cell 133, 1106–1117 (2008). 18555785 10.1016/j.cell.2008.04.043

[b24] Ku M. . Genomewide analysis of PRC1 and PRC2 occupancy identifies two classes of bivalent domains. PLoS Genet. 4, e1000242 (2008). 18974828 10.1371/journal.pgen.1000242 PMC2567431

[b25] Wu H. . Dual functions of Tet1 in transcriptional regulation in mouse embryonic stem cells. Nature 473, 389–393 (2011). 21451524 10.1038/nature09934 PMC3539771

[b26] Visel A., Minovitsky S., Dubchak I. & Pennacchio L. A. VISTA Enhancer Browser--a database of tissue-specific human enhancers. Nucleic Acids Res. 35, D88–D92 (2007). 17130149 10.1093/nar/gkl822 PMC1716724

[b27] Feng B. . Reprogramming of fibroblasts into induced pluripotent stem cells with orphan nuclear receptor Esrrb. Nat. Cell Biol. 11, 197–203 (2009). 19136965 10.1038/ncb1827

[b28] Fischedick G. . Zfp296 is a novel, pluripotent-specific reprogramming factor. PloS ONE 7, e34645 (2012). 22485183 10.1371/journal.pone.0034645 PMC3317644

[b29] Stormo G. D. DNA binding sites: representation and discovery. Bioinformatics 16, 16–23 (2000). 10812473 10.1093/bioinformatics/16.1.16

[b30] Ho Sui S. J. . oPOSSUM: identification of over-represented transcription factor binding sites in co-expressed genes. Nucleic Acids Res. 33, 3154–3164 (2005). 15933209 10.1093/nar/gki624 PMC1142402

[b31] Nagy A. & Gertsenstein M. *Manipulating the Mouse Embryo: A Laboratory Manual* (Cold Spring Harbor Press, (2003).

[b32] O'Geen H., Echipare L. & Farnham P. J. inEpigenetics Protocols 791,265–286Humana Press (2011). 10.1007/978-1-61779-316-5_20 PMC4151291 21913086

[b33] Gaspar-Maia A. . Chd1 regulates open chromatin and pluripotency of embryonic stem cells. Nature 460, 863–868 (2009). 19587682 10.1038/nature08212 PMC3891576

[b34] Langmead B., Trapnell C., Pop M. & Salzberg S. L. Ultrafast and memory-efficient alignment of short DNA sequences to the human genome. Genome Biol. 10, R25 (2009). 19261174 10.1186/gb-2009-10-3-r25 PMC2690996

[b35] Krueger F. & Andrews S. R. Bismark: a flexible aligner and methylation caller for bisulfite-Seq applications. Bioinformatics 27, 1571–1572 (2011). 21493656 10.1093/bioinformatics/btr167 PMC3102221

[b36] Zhang Y. . Model-based analysis of ChIP-Seq (MACS). Genome Biol. 9, R137 (2008). 18798982 10.1186/gb-2008-9-9-r137 PMC2592715

[b37] Wells C. A. . Stemformatics: visualisation and sharing of stem cell gene expression. Stem Cell Res. 10, 387–395 (2013). 23466562 10.1016/j.scr.2012.12.003

