# Supplementary material for: An epigenomic roadmap to induced pluripotency reveals DNA methylation as a reprogramming modulator
Source: Nat Commun. 2014 Dec 10;5:5619. doi: 10.1038/ncomms6619 (PMC4284806; doi:10.1038/ncomms6619)
Supplement: Supplementary Information — Supplementary Figures 1-6 and Supplementary Tables 1-5 [file ncomms6619-s1.pdf]

Supplementary Information
for
“An epigenomic roadmap to induced pluripotency reveals DNA methylation as a reprogramming modulator”

Included in this Supplementary Information file:

Supplementary Figures 1 – 6

Supplementary Tables 1 – 5

All data used can also be visualised at
http://www.stemformatics.org/projects/project_grandiose

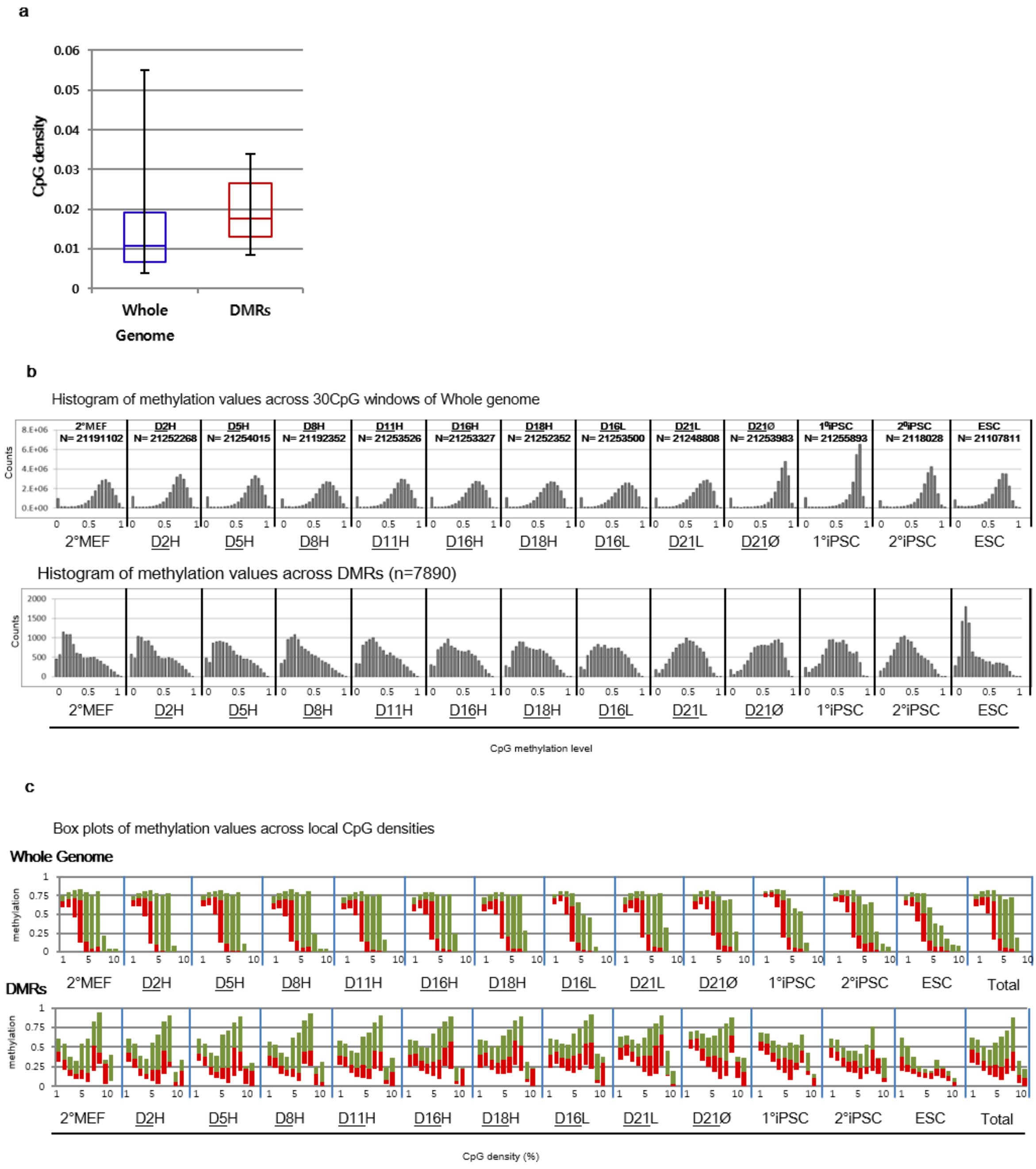

Supplementary Fig. 1 | General features of DNA methylation on whole genome and DMRs. a, Boxplots of CpG density on whole genome and DMRs. b, Histograms of methylation values across 30 CpG windows of whole genome and across DMRs for each sample. n is number of windows for each stage. c, Boxplots of methylation values across local CpG densities. Edges of green and red boxes indicate the 75th and 25th percentile, respectively. Boundary lines of red and green boxes indicate median.

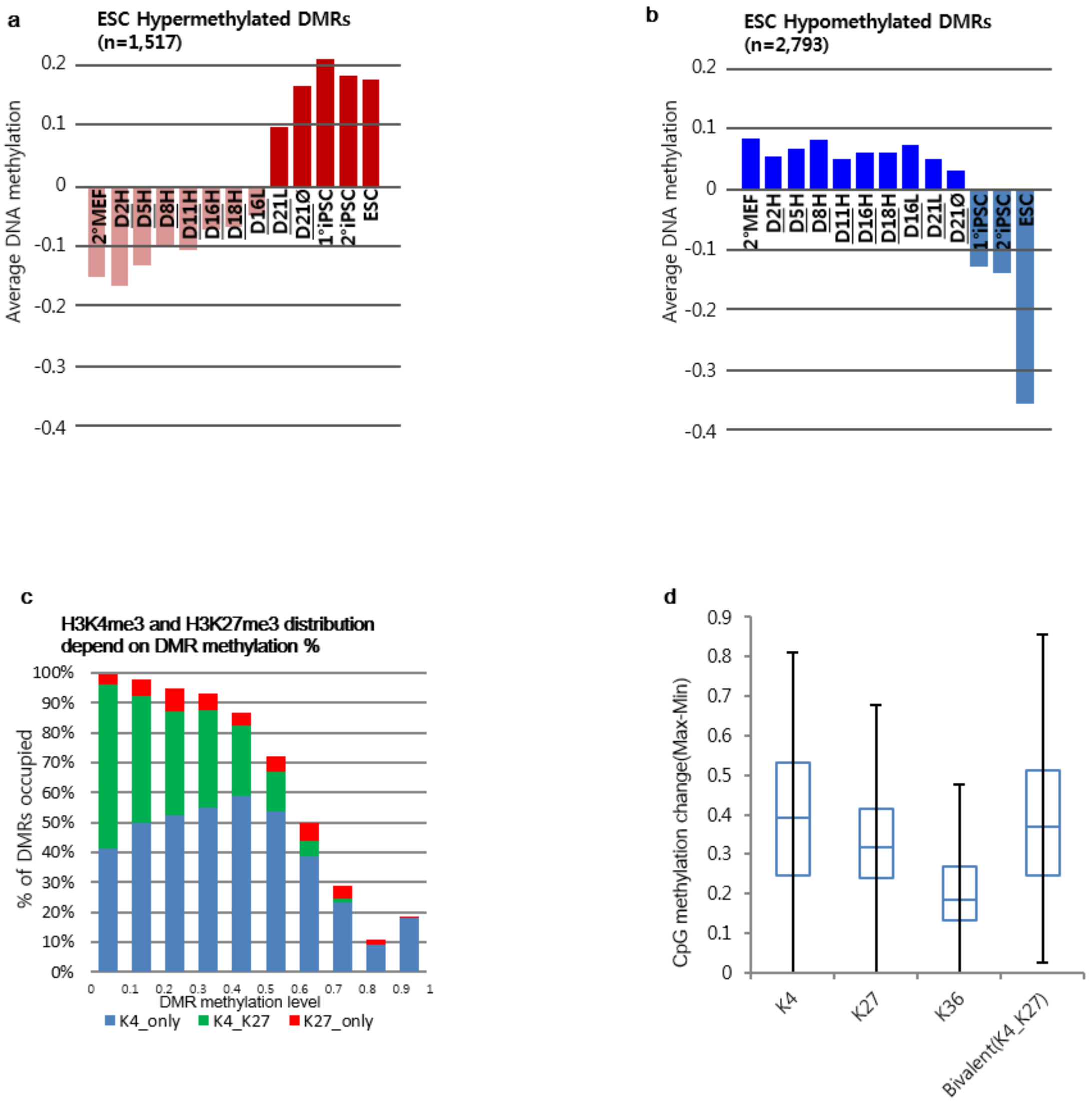

Supplementary Fig. 2 | General features of DNA methylation change and relationship between DNA methylation level and histone modification. a, Average methylation levels for ESC Hyper-DMRs. b, Average methylation levels for ESC Hypo-DMRs. c, H3K4me3 and H3K27me3 occupancy for each DMR methylation level in all samples. d, Boxplots of CpG methylation change within each histone mark cluster (CpG methylation change was calculated by subtracting minimum CpG methylation level from maximum CpG methylation level in each histone mark cluster; see Methods)

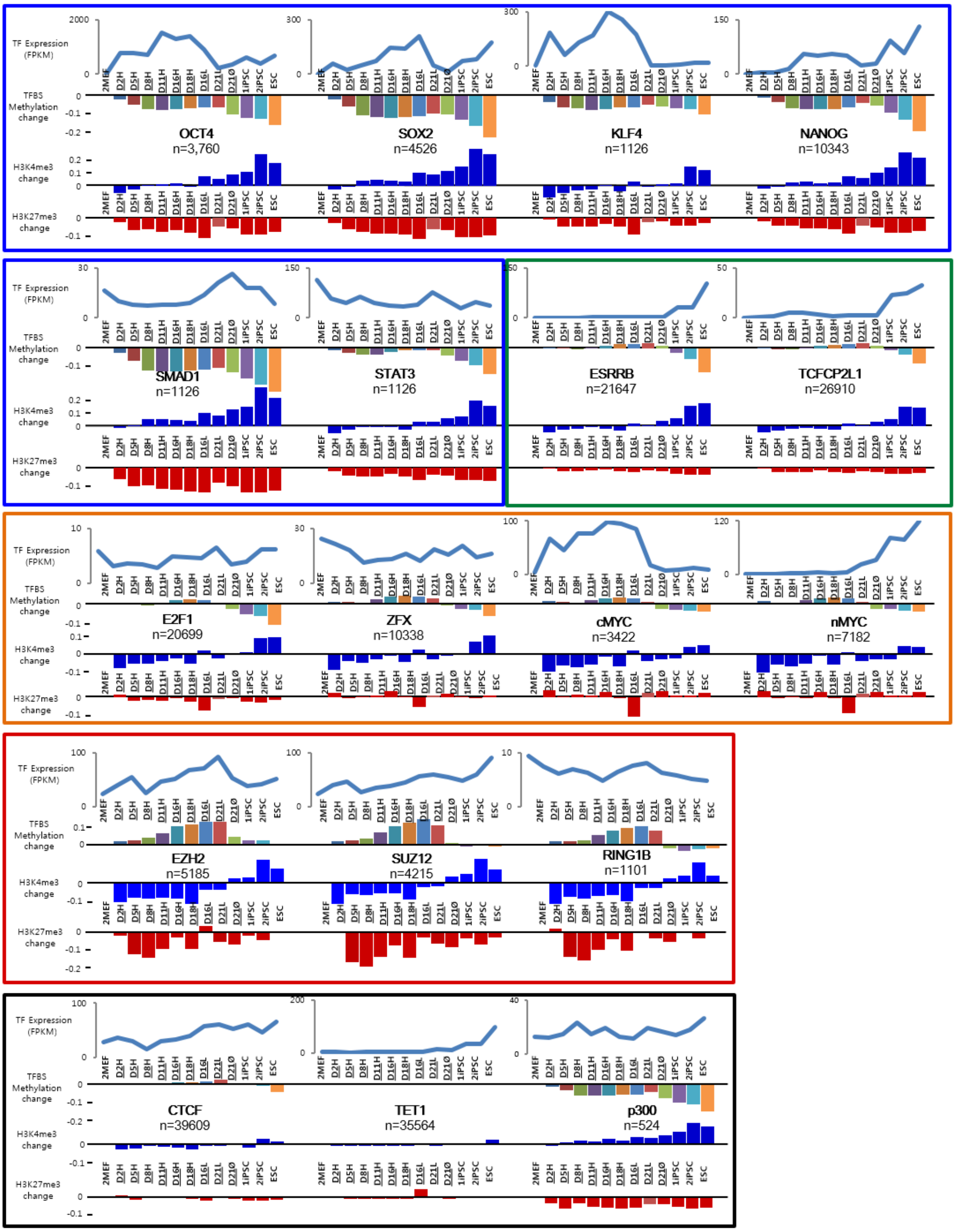

Supplementary Fig. 3 | RNA expression level (FPKM) of transcription factors (line plots), average DNA methylation change (upper bar plots) versus 2MEF, average H3K4me3 change (blue bar plots) and average H3K27me3 change (red bar plots) at binding sites of each transcription factor. Transcriptionally active genes during high-dox treatment (blue box), transcriptionally silent genes during high-dox treatment (green box), polycomb repressive complexes (PRCs) (red box), and others (black box) are shown.

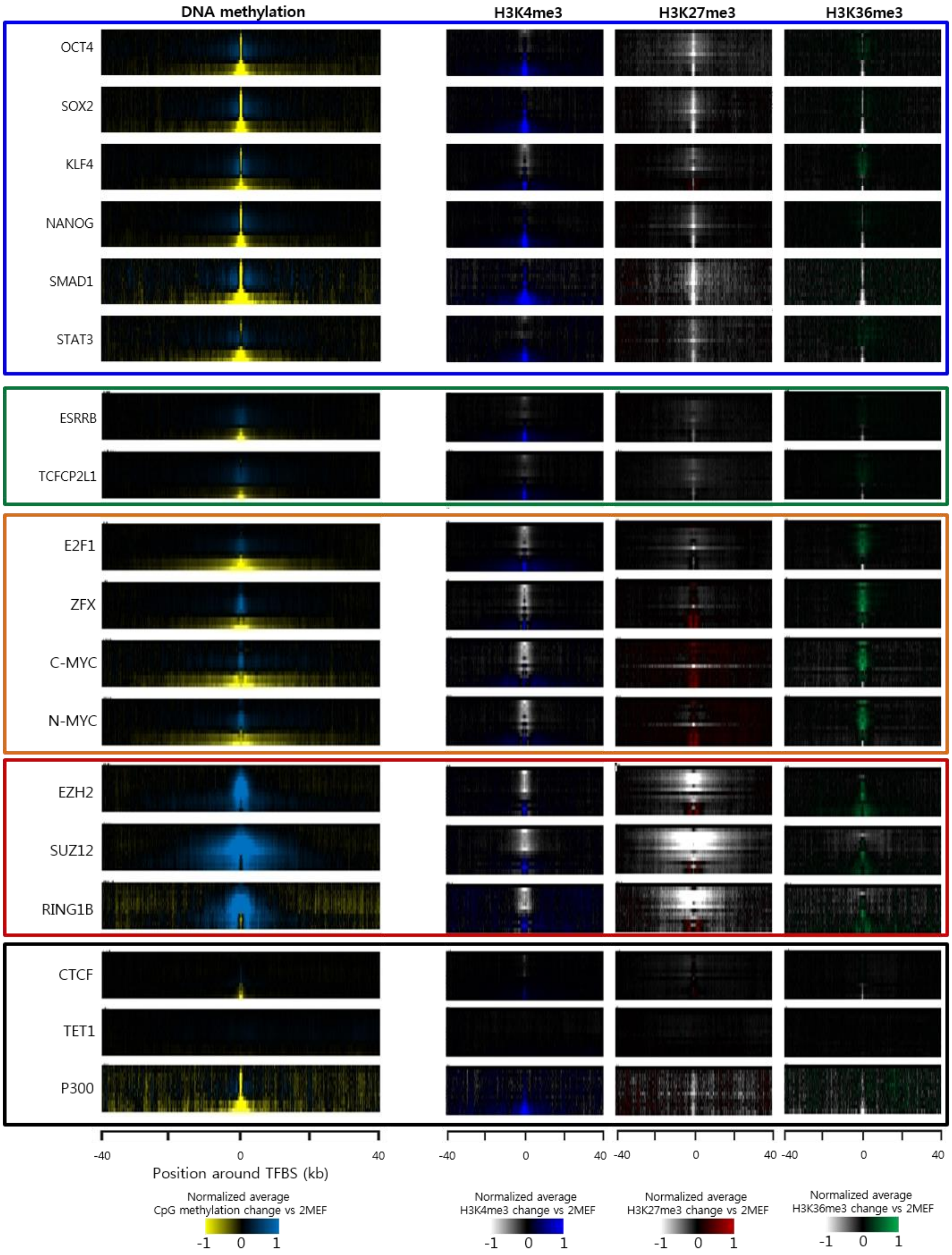

Supplementary Fig. 4 | Average DNA methylation change (left), average H3K4me3 change (middle) and average H3K27me3 change (right) in the 80kb neighborhood of transcription factor binding sites. For each transcription factor, the 13 samples are arranged vertically in standard order. Transcription factors are grouped (coloured boxes) as in Supplementary Fig. 3.

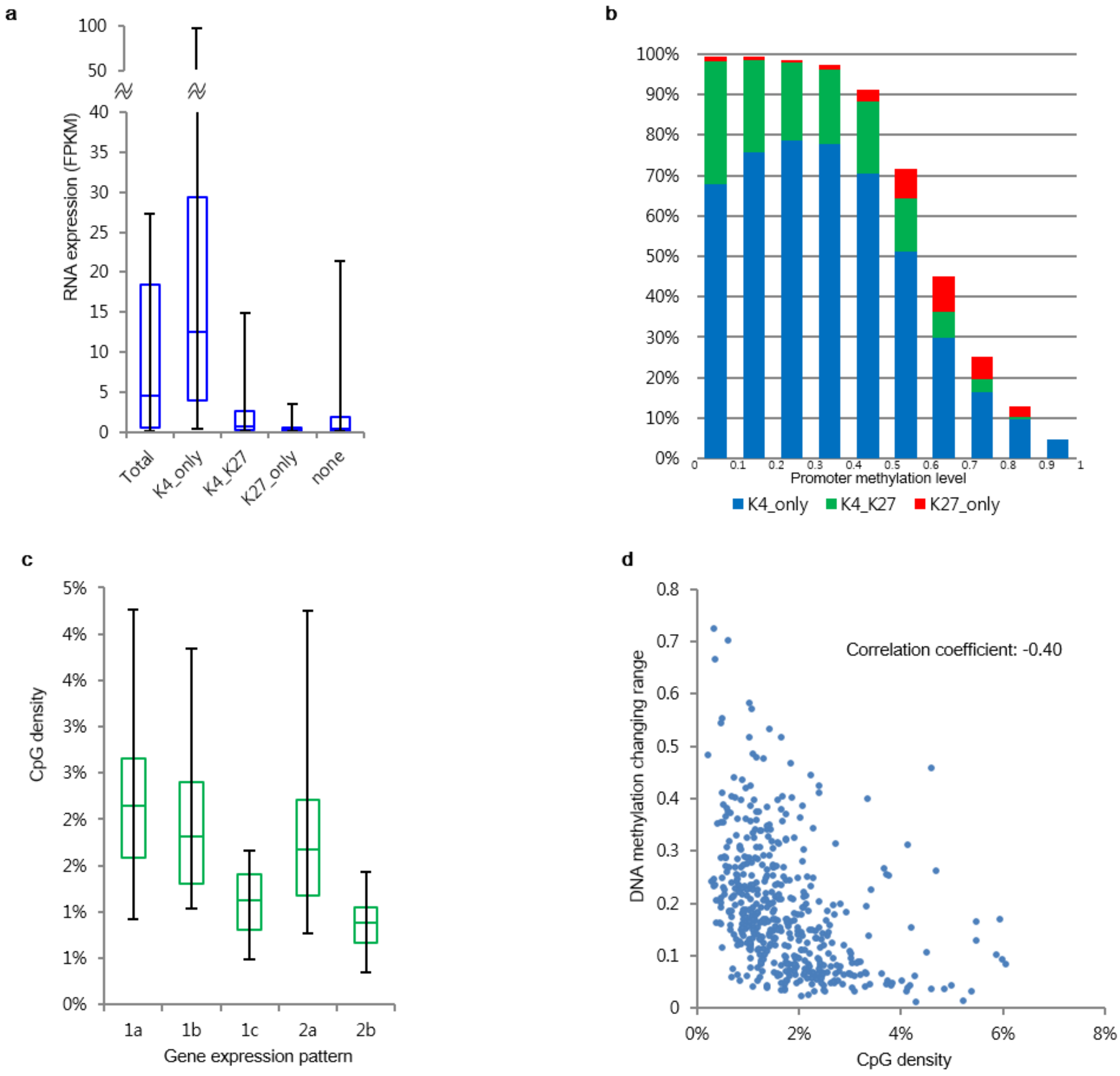

Supplementary Fig. 5 | Relationship between DNA methylation, histone modification, RNA expression, and CpG density. a, Boxplots of expression levels for genes with different histone mark occupancy in promoter regions. b, Occupancy of H3K4me3 and H3K27me3 marks in promoters, for each methylation level of promoters. c, Boxplots of CpG density in promoters of genes in each expression group as described in Fig. 3a. d, Relationship between promoter CpG density and range of change in DNA methylation levels across samples in all genes.

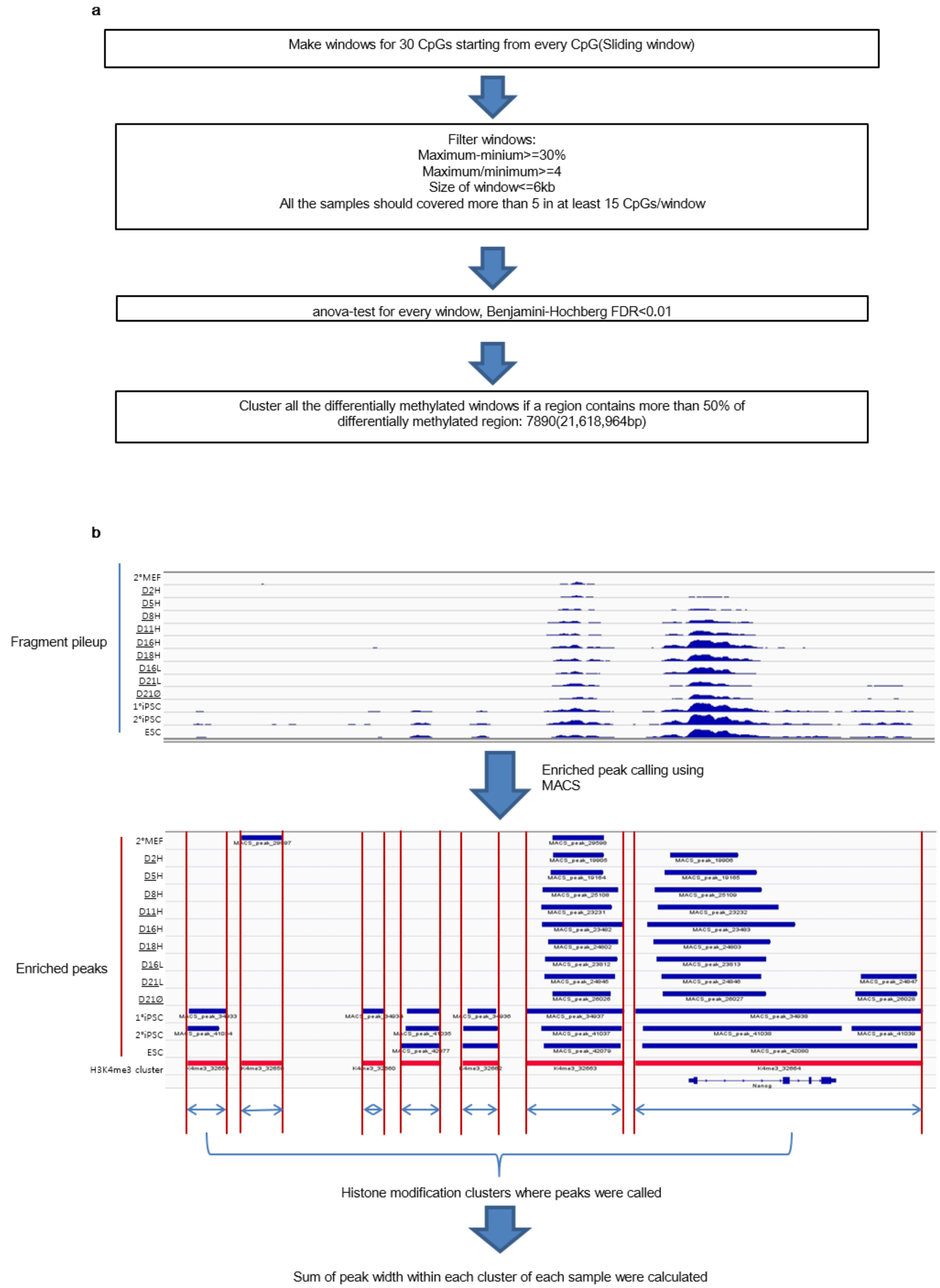

Supplementary Fig. 6 | Scheme for identifying differentially methylated regions (a) and histone mark clusters (b).

Supplementary Table 1. H3K4me3 and H3K27me3 occupancy in DMRs depend on methylation level

		2°MEF	D2H	D5H	D8H	D11H	D16H	D18H	D16L	D21L	D21Ø	1°iPSC	2°iPSC	ESC	Sum
DMRs methylation % <=0.3	K4me3 only	2055 (45.9%)	2143 (45.0%)	2170 (49.9%)	2214 (55.3%)	2025 (48.2%)	1534 (43.7%)	1524 (45.7%)	1264 (45.0%)	540 (43.4%)	852 (48.6%)	1322 (48.9%)	1569 (51.6%)	3544 (62.1%)	22756 (49.6%)
	Both K4/K27me3	415 (9.3%)	475 (10.0%)	343 (7.9%)	232 (5.8%)	339 (8.1%)	371 (10.6%)	362 (10.9%)	350 (12.5%)	17 (1.4%)	3 (0.2%)	0 (0.0%)	0 (0.0%)	3 (0.1%)	18808 (41.0%)
	K27me3 only	1910 (42.7%)	1846 (38.8%)	1572 (36.2%)	1409 (35.2%)	1650 (39.3%)	1494 (42.5%)	1281 (38.4%)	1110 (39.5%)	659 (53.0%)	890 (50.8%)	1378 (50.9%)	1472 (48.4%)	2137 (37.5%)	2910 (6.3%)
	no K4me3 or K27me3	93 (2.1%)	295 (6.2%)	260 (6.0%)	152 (3.8%)	187 (4.5%)	115 (3.3%)	165 (5.0%)	83 (3.0%)	28 (2.3%)	8 (0.5%)	5 (0.2%)	2 (0.1%)	20 (0.4%)	1413 (3.1%)
	Total	4473	4759	4345	4007	4201	3514	3332	2807	1244	1753	2705	3043	5704	45887
DMRs methylation % >=0.7	K4me3 only	90 (8.3%)	67 (7.1%)	78 (7.8%)	107 (10.2%)	74 (7.8%)	87 (8.4%)	95 (9.1%)	113 (10.3%)	447 (45.9%)	470 (40.2%)	94 (17.8%)	197 (37.6%)	29 (8.7%)	1948 (16.6%)
	Both K4/K27me3	3 (0.3%)	2 (0.2%)	0 (0.0%)	3 (0.3%)	2 (0.2%)	4 (0.4%)	5 (0.5%)	9 (0.8%)	8 (0.8%)	16 (1.4%)	18 (3.4%)	23 (4.4%)	7 (2.1%)	100 (0.9%)
	K27me3 only	28 (2.6%)	13 (1.4%)	12 (1.2%)	10 (1.0%)	11 (1.2%)	17 (1.6%)	14 (1.3%)	21 (1.9%)	12 (1.2%)	50 (4.3%)	64 (12.1%)	49 (9.4%)	32 (9.6%)	333 (2.8%)
	no K4me3 or K27me3	965 (88.9%)	868 (91.4%)	904 (90.9%)	931 (88.6%)	856 (90.8%)	925 (89.5%)	932 (89.1%)	953 (87.0%)	507 (52.1%)	634 (54.2%)	353 (66.7%)	255 (48.7%)	266 (79.6%)	9349 (79.7%)
	Total	1086	950	994	1051	943	1033	1046	1096	974	1170	529	524	334	11730

Supplementary Table 2. Correlation coefficient between gene expression and eipgenomic changes

Relationship with Total Gene(n=37412)	H3K4me3	DMR	H3K27me3	H3K36me3	Promoter CpG methylation	Gene body CpG methylation
**Containing number**	20816	4320	13497	14198	37412	37412
**Average correlation**	0.25	-0.25	-0.14	0.23	-0.12	-0.06
**Strong correlation (R>=0.5)**	6226	206	511	3682	1525	2104
**Strong anti-correlation (R<=-0.5)**	414	1298	1691	226	5081	3838

Supplementary Table 3. Correlation coefficient between differentially expressed genes and epigenomic changes

Relationship with DEG(n=547)	H3K4me3	DMR	H3K27me3	H3K36me3	CpG methylation	Gene body CpG methylation
**Containing number**	438	180	315	283	547	547
**Average correlation**	0.57	-0.40	-0.29	0.54	-0.13	-0.07
**Strong correlation (R>=0.5)**	300	7	14	165	48	39
**Strong anti-correlation (R<=-0.5)**	5	83	102	2	119	94

Supplementary Table 4. H3K4me3 and H3K27me3 occupancy in promoters depend on methylation level

		**2°MEF**	**D2H**	**D5H**	**D8H**	**D11H**	**D16H**	**D18H**	**D16L**	**D21L**	**D21Ø**	**1°iPSC**	**2°iPSC**	**ESC**	Total
Gene Promoters methylation % <=0.3	K4me3 only	6026 (62.6%)	6736 (66.2%)	7144 (72.4%)	7143 (74.7%)	7128 (69.6%)	6471 (63.7%)	6699 (65.4%)	6444 (64.9%)	5929 (67.3%)	5756 (69.6%)	5535 (62.0%)	5517 (60.6%)	6809 (64.6%)	83337 (66.4%)
	Both K4/K27me3	3351 (34.8%)	3047 (29.9%)	2434 (24.7%)	2154 (22.5%)	2679 (26.2%)	3120 (30.7%)	2897 (28.3%)	2798 (28.2%)	2551 (28.9%)	2374 (28.7%)	3312 (37.1%)	3498 (38.4%)	3614 (34.3%)	37829 (30.2%)
	K27me3 only	105 (1.1%)	190 (1.9%)	103 (1.0%)	69 (0.7%)	77 (0.8%)	99 (1.0%)	99 (1.0%)	83 (0.8%)	5 (0.1%)	6 (0.1%)	5 (0.1%)	7 (0.1%)	6 (0.1%)	854 (0.7%)
	no K4me3 or K27me3	143 (1.5%)	202 (2.0%)	190 (1.9%)	190 (2.0%)	352 (3.4%)	464 (4.6%)	554 (5.4%)	610 (6.1%)	330 (3.7%)	138 (1.7%)	71 (0.8%)	76 (0.8%)	111 (1.1%)	3431 (2.7%)
	Total	9625	10175	9871	9556	10236	10154	10249	9935	8815	8274	8923	9098	10540	125451
Gene Promoters methylation % >=0.7	K4me3 only	1231 (9%)	754 (7%)	801 (7%)	1156 (10%)	821 (9%)	877 (10%)	884 (11%)	961 (11%)	1307 (11%)	1628 (10%)	1642 (9%)	2005 (11%)	1209 (8%)	15276 (10%)
	Both K4/K27me3	109 (1%)	39 (0%)	48 (0%)	49 (0%)	41 (0%)	49 (1%)	33 (0%)	52 (1%)	119 (1%)	308 (2%)	362 (2%)	434 (2%)	211 (1%)	1854 (1%)
	K27me3 only	1428 (11%)	452 (4%)	486 (4%)	334 (3%)	203 (2%)	236 (3%)	127 (2%)	262 (3%)	440 (4%)	1587 (9%)	1059 (6%)	1075 (6%)	555 (4%)	8244 (5%)
	no K4me3 or K27me3	10553 (79%)	9247 (88%)	9798 (88%)	10413 (87%)	7749 (88%)	7323 (86%)	7207 (87%)	7308 (85%)	9749 (84%)	13609 (79%)	15314 (83%)	14209 (80%)	12327 (86%)	134806 (84%)
	Total	13321	10492	11133	11952	8814	8485	8251	8583	11615	17132	18377	17723	14302	160180

Supplementary Table 5. Gene separation strategy based on expression

			Sample_context(X_FPKM<=1.5, O_FPKM>=5)					Number of genes
			2°MEF	D16H	D18H	2°iPSC	ESC	
ESC like	Activation	in F-class and ESC-like cells (1a)	X	O	O	O	O	87
		Only in ESC-like cells (1b,1c)	X	X	X	O	O	93
	Repression	in F-class and ESC-like cells (2a, 2b)	O	X	X	X	X	221
		Only in ESC-like cells	O	O	O	X	X	14
Fail	Activation		X	X	X	X	O	47
	Repression		O	O	O	O	X	9
F-class specific	Activation (3a)		X	O	O	X	X	41
	Repression (3b)		O	X	X	O	O	35
